# RNAi-Mediated Gene Silencing in a Gonad Organ Culture to Study Sex Determination Mechanisms in Sea Turtle

**DOI:** 10.3390/genes4020293

**Published:** 2013-06-07

**Authors:** Itzel Sifuentes-Romero, Horacio Merchant-Larios, Sarah L. Milton, Norma Moreno-Mendoza, Verónica Díaz-Hernández, Alejandra García-Gasca

**Affiliations:** 1 Laboratory of Molecular Biology, Research Centre for Nutrition and Development (CIAD), Avenida Sábalo-Cerritos s/n, Mazatlán, Sinaloa 82010, Mexico; E-Mail: itzel.sifuentes@ciad.mx; 2 Institute for Biomedical Research, Universidad Nacional Autónoma de México (UNAM), Mexico City 04510, Mexico; E-Mails: merchant@unam.mx (H.M.-L.); angelica@biomedicas.unam.mx (N.M.-M.); 3 Department of Biological Sciences, Florida Atlantic University, 777 Glades Road, Boca Raton, FL 33431, USA; E-Mail: smilton@fau.edu; 4 Department of Embryology, School of Medicine, Universidad Nacional Autónoma de México (UNAM), Mexico City 04510, Mexico; E-Mail: roveazdih@yahoo.com.mx

**Keywords:** *Sox9*, *Amh*, RNAi, *Lepidochelys olivacea*, gonad culture

## Abstract

The autosomal *Sry*-related gene, *Sox9*, encodes a transcription factor, which performs an important role in testis differentiation in mammals. In several reptiles, *Sox9* is differentially expressed in gonads, showing a significant upregulation during the thermo-sensitive period (TSP) at the male-promoting temperature, consistent with the idea that SOX9 plays a central role in the male pathway. However, in spite of numerous studies, it remains unclear how SOX9 functions during this event. In the present work, we developed an RNAi-based method for silencing *Sox9* in an *in vitro* gonad culture system for the sea turtle, *Lepidochelys olivacea*. Gonads were dissected as soon as the embryos entered the TSP and were maintained in organ culture. Transfection of siRNA resulted in the decrease of both *Sox9* mRNA and protein. Furthermore, we found coordinated expression patterns for *Sox9* and the anti-Müllerian hormone gene, *Amh*, suggesting that SOX9 could directly or indirectly regulate *Amh* expression, as it occurs in mammals. These results demonstrate an *in vitro* method to knockdown endogenous genes in gonads from a sea turtle, which represents a novel approach to investigate the roles of important genes involved in sex determination or differentiation pathways in species with temperature-dependent sex determination.

## 1. Introduction

Several species of reptiles apparently lack sex chromosomes and display temperature-dependent sex determination (TSD). In these species, the differentiation of gonads into ovaries or testes depends on the incubation temperature of the eggs during a critical period of embryonic development known as the thermo-sensitive period (TSP) [1,2]. In mammals, sex determination depends on the *Sry* gene [3], but no *Sry* homologue has been found in reptiles. Nevertheless homologues of several other mammalian sex-determining genes have been identified [4,5,6]. Among them, the autosomal *Sry*-related gene, *Sox9*, has been implicated in testis differentiation in birds [7,8] and reptiles [9,10,11,12,13]. *Sox9* encodes a transcriptional activator necessary for mammalian testis development [14,15] and is a direct target of SRY [16]; since SOX9 has the ability to virtually replace the action of SRY in mammals [17], it may play a critical role in testis development in organisms displaying TSD.

In the American alligator, *Alligator mississippiensis*, basal levels of *Sox9* expression have been observed early in the TSP at male-promoting temperature (MPT); expression was then upregulated at the end of the TSP, during testis differentiation, whereas at female-promoting temperature (FPT), *Sox9* was expressed at basal levels at all stages [9]. Since upregulation occurred after sex determination and coincided with the differentiation of the testis, the authors concluded that this gene plays an important role in testis differentiation, but not in sex determination. In contrast, in the red-eared slider turtle, *Trachemys scripta*, *Sox9* is expressed early in undifferentiated gonads at both MPT and FPT and then becomes restricted to the developing testis at the end of the TSP [11,12]. In the olive ridley sea turtle, *Lepidochelys olivacea*, SOX9 protein is present during the TSP in undifferentiated gonads of embryos incubated at both MPT and FPT. However, at the middle of the TSP, SOX9 remains present at MPT, while at FPT, it is downregulated. At the onset of gonadal differentiation, SOX9 protein is still present at MPT, but is not detected at FPT [10,18,19]. It has also been shown that SOX9 responds to temperature in cultured gonads exposed to sex-reversing temperature shifts in either direction (MPT to FPT and FPT to MPT) in both *L. olivacea* [20] and *T. scripta* [21].

In humans and mice, SOX9 is known to activate transcription of the anti-Müllerian hormone gene (*Amh*), which is critical for regression of the female structures derived from the Müllerian ducts [22], but not necessary for sex determination [23]. Consistent with this role, in *T. scripta* [11,12] and *L. olivacea* [24], it has been found that *Sox9* expression precedes that of *Amh*. In contrast, in chicken and alligator, *Sox9* follows the expression of *Amh*, suggesting that SOX9 is not required for the activation of *Amh* [25,26]. Thus, the role of SOX9 in sex determination/differentiation in reptiles remains elusive.

Gene silencing has been used to study the function of particular genes in different biological systems or backgrounds. Post-transcriptional gene silencing (PTGS) involves the repression of gene expression through specific mRNA degradation, representing an attractive strategy, because it has the potential to silence gene expression of any gene at any developmental stage. RNA interference (RNAi) has been widely used in PTGS approaches to elucidate gene function in several species [27]. While RNAi studies typically have been conducted in monolayer cell culture and *in vivo*, recently, the RNAi methodology has been adapted for use in organ culture of mouse kidney rudiments and ovaries [28,29].

In the present study, we developed an RNAi-based method to silence *Sox9* in a turtle gonad culture system for *L. olivacea*. We hypothesized that if SOX9 is required for testis determination/differentiation, then *Sox9* knockdown at MPT (26 °C) would result in gonad feminization evidenced by the expression of female-specific genes. Also, if SOX9 acts upstream of *Amh*, the latter should be downregulated after *Sox9* knockdown. To evaluate the expression of a female-specific gene, we chose P-450 Aromatase (*Cyp19a*) as a potential candidate, since it is upregulated in the female gonad at the end of the TSP. The present study represents a novel approach to elucidate the function of genes involved in sex determination or differentiation in organisms displaying TSD.

## 2. Materials and Method

### 2.1. Egg Incubation

Freshly laid eggs of *L. olivacea* were obtained from La Escobilla beach in Oaxaca, Mexico, and transported to the Instituto de Investigaciones Biomédicas, UNAM, in Mexico City. Upon arrival, the eggs were divided into two batches and incubated at 26 °C (MPT) and 33 °C (FPT) in trays of moistened vermiculite.

### 2.2. Design of Turtle Specific Sox9 siRNAs

Using the Invitrogen siRNAs designer tool, two specific *Sox9* siRNAs were designed from the transactivation domains of the *L. olivacea Sox9* (GQ258676) sequence: Lo-siRNA(1) 5'-CAG CAU GAG UGA GGU UCA CUC UCC A-3', Lo-siRNA(2) 5'-UAG AGA CCU UUG ACG UAA AUG AGU U-3'. Both siRNAs were transfected together into the gonads. Additionally, we designed a scrambled control sequence: Lo-siRNA(cs) 5'-AGU CCA AGU UAU GCG UAA AGA GUU-3'.

### 2.3. Gonad Culture and Transfection

In order to develop a protocol to culture the gonads and knockdown Sox9, we worked first with *T. scripta* embryos as a preliminary approach in Florida Atlantic University, Boca Raton, FL, USA, according to Davies *et al.* [28] and Nayak *et al.* [30], and then, we adapted the protocol to *L. olivacea*. Since *Sox9* expression has been detected prior the TSP at MPT [18], eggs were incubated until Miller’s stage (st) 23 [31]. Adrenal-kidney-gonad (AKG) complexes were dissected and floated in L-15 medium (Invitrogen, Life Technologies, Grand Island, NY, USA). The gonads were cultured at 26 °C (MPT), according to Moreno-Mendoza *et al.* [20]. We designed a dose curve with three different doses of siRNAs containing equimolar amounts of both siRNAs. Transfections were performed according to the Lipofectamine protocol from Invitrogen; briefly, the transfection mix was prepared using 100, 300 and 600 pmol of siRNA (both sequences) and 40 µL of Lipofectamine 2000 (Invitrogen, Life Technologies, Grand Island, NY, USA) in 600 µL of Opti-MEM (Invitrogen, Life Technologies, Grand Island, NY, USA). Similarly, the control transfection mix was prepared without siRNA and 40 µL of Lipofectamine 2000 in 600 µL of Opti-MEM. The supplemented media was removed from each well, and the transfection mix was applied directly to organ cultures. The transfected gonads were incubated at 26 °C for 72 hours.

Gonads incubated *in ovo* at the same temperature and stage (MPT st 23) were used as positive controls for Sox9 protein and mRNA expression assays. Since it is known that Sox9 is downregulated at FPT after st 27 [10,18], we decided to use gonads incubated *in ovo* at st 29 as negative controls. We also included three experimental controls *in vitro* (MPT st 23): CO (gonads cultured only with Opti-MEM); CL (gonads cultured with Lipofectamin and Opti-MEM); and CS (gonads transfected with the scrambled control sequence at a concentration of 300 pmol). Sox9 was examined at the protein and mRNA levels. Also, cell proliferation assays were performed by labeling with bromodeoxyuridine (BrdU), according to the manufacturer’s instructions (Roche Diagnostics, Mexico City, Mexico).

### 2.4. Immunofluorescence

SOX9 and cytokeratin (epithelial cells marker) were sequentially detected in 10 µm frozen sections from *L. olivacea*’s gonads. These were washed in PBS and incubated in 10 mM sodium citrate, pH 6, at 85 °C for 45 minutes for antigen retrieval. The slides were blocked with 5% inactivated horse serum in PBS-T (PBS/0.5% Triton) for 1 hour and subsequently incubated overnight first with rabbit anti-turtle SOX9 primary antibody diluted 1:150 at 4 °C [32]. After washing with PBS, sections were incubated with goat Cy5-conjugated anti-rabbit secondary antibody (Millipore, Mexico City, Mexico) (for 40 minutes at room temperature), then blocked with 1% horse serum in PBST for 30 minutes and incubated overnight with a mouse anti-Pan Cytokeratin Plus (AE1/AE3 + 8/18) antibody (Biocare Medical, Mexico City, Mexico) diluted 1:100 at 4 °C. Sections were then incubated with Alexa Fluor 488-conjugated anti-mouse secondary antibody (Invitrogen, Molecular Probes, Grand Island, NY, USA) for 40 minutes at room temperature. Cell nuclei were stained with TOTO-3iodide (Invitrogen, Molecular Probes, Grand Island, NY, USA). Frozen sections of gonads of embryos incubated at FPT (stage 29) and MPT (st 23) *in ovo* were included as negative and positive controls, respectively, for the presence of Sox9 protein. Additionally, proliferative cells were detected using an anti-BrdU antibody (Roche Diagnostics, Mexico City, Mexico); this immunofluorescence required pre-treatment with 2N HCl for 30 minutes at 37 °C prior to blocking. The images were captured and processed with a confocal Pascal LSM5-Zeiss microscope.

### 2.5. Quantitative PCR

Total RNA was extracted using Trizol reagent (Invitrogen, Accesolab, Mexico City, Mexico), followed by DNAse I treatment. cDNA was reverse-transcribed using random primers and the MMLV reverse transcriptase (Promega, Uniparts, Mexico City, Mexico). Primers used to assay gene expression were designed from the transactivation domain of *L. olivacea Sox9* (Forward: 5'-GGG AAG ACA ACC GCC ACA CAT TG-3'; Reverse: 5'-GGC GTG CTG CTG ATC CCA TA-3'), from *L. olivacea Amh* (Forward: 5'-AGA AGT TCC AGG CTG TCA TCC A-3'; Reverse: 5'-ACC TTC CTC TTC TCC TGC CAG T-3') and from *L. olivacea* Aromatase (Forward: 5'-TGA AAA ACT GGA AGA CCA CAT GGA-3'; Reverse: 5'-TTC CAC TTT AGG GTG CTC TGC AAT-3'), amplifying fragments of 200, 217 and 181 bp, respectively. Gene expression was quantified with a SmartCycler (Cepheid, BioSelec, Mexico City, Mexico) using EvaGreen^®^ (BioRad, Mexico City, Mexico). Individual samples were analyzed in duplicate, and β-actin was used as internal control [18]. PCR conditions were as follows: one cycle at 95 °C 30 seconds, 40 cycles at 95 °C, 5 seconds, and 60 °C, 20 seconds. Relative gene expression levels were calculated through the comparative ΔCT method according to Livak and Schmittgen [33]. ΔCT values were converted to the linear form using the 2^−^^ΔCT^ equation [33]. One-way ANOVA coupled to an all pairwise multiple comparison analysis (Holm-Sidak test) was performed using 2^−^^ΔCT^ data to find statistical differences in gene expression between groups (*p* < 0.05).

## 3. Results and Discussion

In order to develop a protocol to knockdown *Sox9* in a gonad organ culture system, we worked first with the non-endangered species, *T. scripta*, based on previous reports [28,30], as a preliminary approach. To optimize conditions, we performed different assays, varying the amount of siRNA and Lipofectamine; also, we tested different culture media and incubation times with siRNA. Once the protocol was established, we adapted it to *L. olivacea*.

### 3.1. Sox9 Protein and mRNA Expression

**Figure 1:**
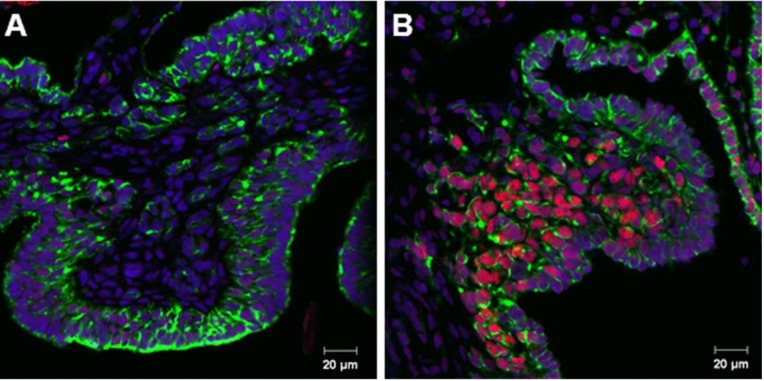
Double immunofluorescence of SOX9 (red) and cytokeratin (green) in gonads from *L. olivacea* in ovo. (**A**) Gonads of embryos incubated at female-promoting temperature (FPT) Miller’s stage (st) 29 and (**B**) male-promoting temperature (MPT) st 23.

Consistent with previous reports of SOX9 expression in *L. olivacea* gonads [10], female gonads (st 29) did not show a SOX9 signal, neither in medullar cords or in the surface epithelium (Figure 1A), whereas male gonads (st 23) showed a positive and specific SOX9 signal in medullar cords, but not in the surface epithelium (Figure 1B).

**Figure 2:**
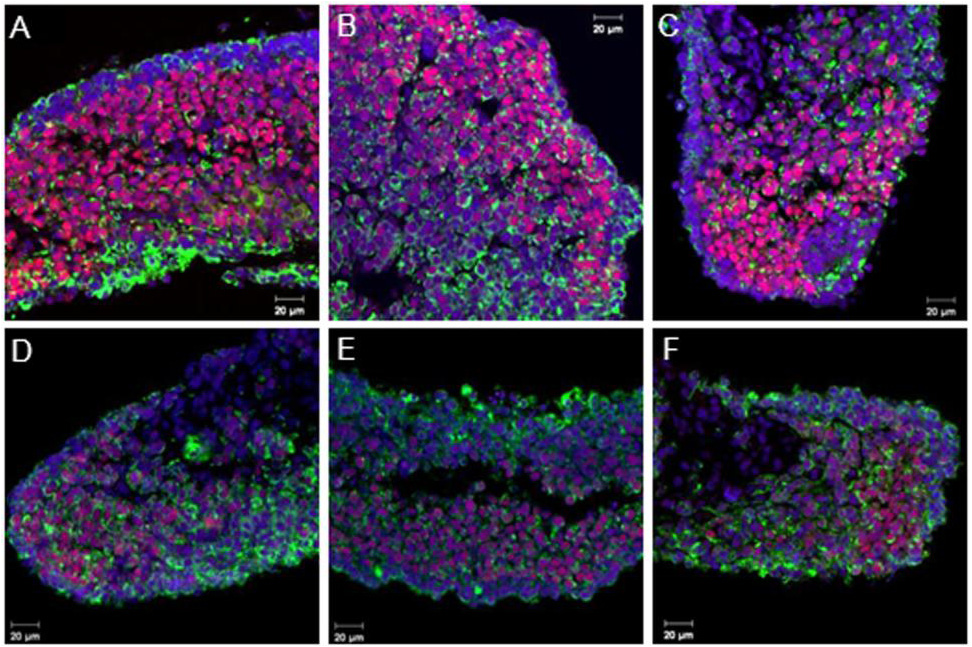
Double immunofluorescence of SOX9 (red) and cytokeratin (green) in gonads from *L. olivacea* cultured with (**A**) Opti-MEM, CO, (**B**) Lipofectamin and Opti-MEM, CL, (**C**) transfected with the scrambled control sequence, CS, and (**D**–**F**) transfected with 100, 300 and 600 pmol of siRNA, respectively. Scale bar = 20 µm.

**Figure 3:**
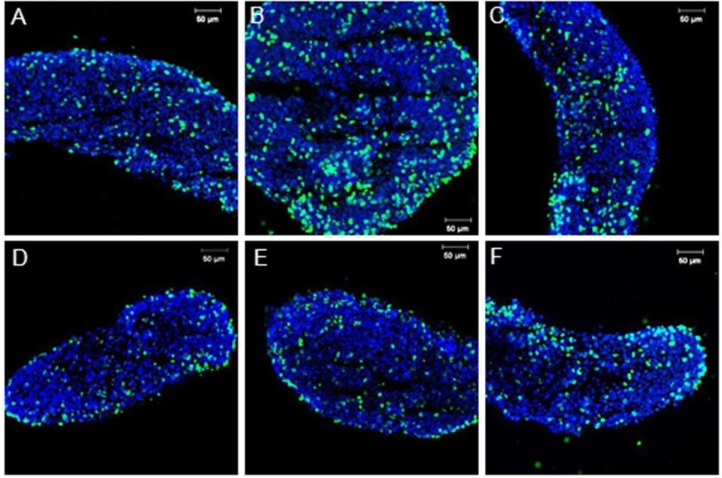
Immunofluorescence of BrdU in experimental controls (**A**) CO, (**B**) CL, (**C**) CS; and (**D**–**F**) gonads transfected with 100, 300 and 600 pmol of siRNA, respectively. Scale bar = 20 µm.

Similar results were shown *in vitro* with the experimental controls in which CO, CL and CS showed a positive SOX9 signal only in medullar cords (Figure 2A–C, respectively). These results suggest that neither the *in vitro* culture media nor the Lipofectamine or the control scrambled siRNA affected SOX9 protein expression. siRNA transfection in *L. olivacea* cultured gonads resulted in a decrease of SOX9 protein in the three concentrations tested (Figure 2D–F). These results, although not quantitative, suggest that *Sox9* expression was (at least partially) blocked. BrdU incorporation in experimental controls (Figure 3A–C) and transfected gonads (Figure 3D–F) seemed similar; although cell death due to toxicity was not assessed in this study, these results (though not quantitative) showed comparable cell proliferation of *Sox9*-expressing cells in all experiments.

**Figure 4:**
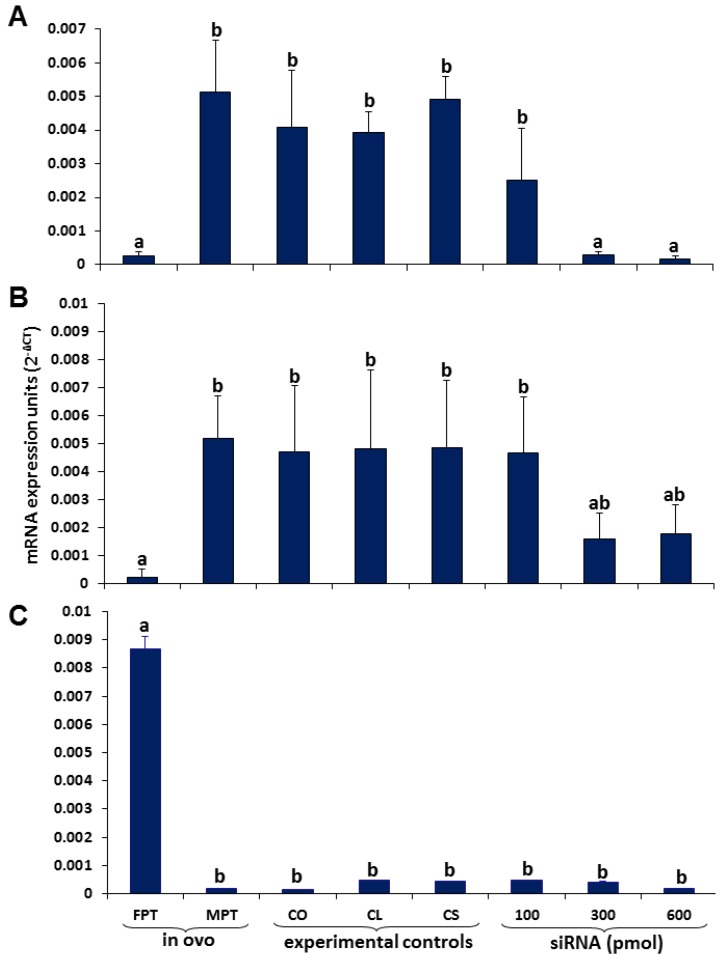
Relative expression of (**A**) *Sox9*, (**B**) *Amh* and (**C**) Aromatase in *L. olivacea* showing a decreased expression of *Sox9* and *Amh* in gonads transfected with 300 and 600 pmol of siRNA. Aromatase expression levels remained low in all treatments, except at FPT. Different letters indicate significant differences in expression levels. n = 3 for each group.

Consistent with our results and with a previous report [18], qPCR analysis showed significantly higher mRNA levels of *Sox9* in gonads incubated *in ovo* at MPT (st 23) than in gonads at FPT (st 29; *p* < 0.001; Figure 4A). *Sox9* expression levels in two of the experimental controls (CO and CL) were slightly lower than those in the *in vivo* control at MPT; nevertheless, the differences were not significant (*p* = 0.22 and 0.442 for CO and CL, respectively); the experimental control, CS, showed similar expression levels to the *in vivo* control incubated at MPT (*p* = 0.819) (Figure 4A). We observed a decrease of *Sox9* mRNA levels in transfected gonads. The decrease observed with the lowest siRNA concentration (100 pmol) was not significant; however, higher concentrations of siRNAs (300 and 600 pmol) were significantly lower than the *in vivo* control at MPT and the three experimental controls (CO, CL and CS) (*p* < 0.001, Figure 4A). Yet, they did not show significant differences between each other (*p* = 0.966), suggesting that 300 pmol was enough to knockdown *Sox9*. Off-target effects were not detected.

Previously, Davies *et al*. [28] adapted this technique in an organ culture system of kidney rudiments of mice to investigate a regulator of renal development, Wt1. A similar study was conducted more recently by Wang *et al*. [29] in which they reduced Hnrnpk mRNA levels using siRNAs in neonatal ovaries of mice in culture. In both studies, the authors not only showed a decrease in the target protein, but also showed a phenotypic effect. Unfortunately, in the present study, we were not able to observe any morphological effect in cultured gonads, probably due to the short time the gonads were maintained in culture; in 72 hours, the gonads must have changed at most from st 23 to st 24 at MPT. Nevertheless, in an effort to detect any effect of *Sox9* silencing in gonad feminization, we decided to evaluate the expression of a presumably SOX9-regulated gene, the Anti-Müllerian Hormone (*Amh*), which is responsible for the regression of the Müllerian duct in the male gonad, and a female-specific gene, P-450 Aromatase, even if previous works indicate that expression levels of the latter increase in the female gonads from other reptiles at the end of the TSP [34], which in *L. olivacea* would be st 25–26 at MPT and st 26–27 at FPT [23].

### 3.2. Coordinated Expression of Sox9 and Amh

It has been suggested that in *T. scripta* and *L. olivacea*, SOX9 regulates the expression of *Amh* as it occurs in mammals [22]. This has been suggested, due to the expression profiles of both genes, higher at MPT than at FPT, during and after the TSP [11,12,18], and, also, due to a pattern observed in their expression levels, in which *Sox9* expression precedes that of *Amh* [12,23]. Consistent with these observations and our results regarding *Sox9* expression, we found higher expression levels of *Amh* in gonads incubated at MPT (st 23) than gonads incubated at FPT (st 29) (*p* < 0.001, Figure 4B) *in ovo*. In the experimental controls, *in vitro Amh* expression levels were similar to those observed in the *in vivo* control incubated at MPT (*p* = 0.582, 0.518 and 0.235 in CO, CL and CS, respectively). Interestingly, we found a decrease of *Amh* mRNA levels in response to *Sox9* siRNA in gonads transfected with 300 and 600 pmol; although maximal decreases were detected at 300 pmol, no significant differences were detected between this concentration and experimental controls (*p* = 0.073, 0.089 and 0.068 for CO, CL and CS, respectively) or between 300 and 600 pmol (*p* = 0.908, Figure 4B). Further investigations are required to determine whether SOX9, directly or indirectly, influences *Amh* expression.

It has been shown that in *L. olivacea*, AMH protein and its transcript are detected in medullar cords of embryos (st 27) incubated at MPT [23], coinciding with the site of SOX9 protein detection [10] and also with *Sox9* mRNA localization [23]. These observations suggest that in *L. olivacea*, the mechanisms mediating male reproductive tract development (including Sertoli cell differentiation and Müllerian duct regression) involve the coordinated action of both genes. First, SOX9 triggers Sertoli cell differentiation and then activates *Amh* expression, leading to the regression of the Müllerian duct as it occurs in mammals [35]. It is worth mentioning that in mammals, AMH is not required for Sertoli cell differentiation, since human individuals harboring a homozygous mutation for the AMH gene show Müllerian duct persistence, but normal Sertoli cells [35].

### 3.3. Aromatase Expression

Aromatase, a CYP450 enzyme, is responsible for the conversion of androgens into estrogens. Previously, it has been shown that both aromatase expression and activity increase at the end of the TSP in gonads incubated at FPT, while at MPT, they remain low, suggesting a role for differential estrogen production in ovarian determination/differentiation in marine and fresh water turtles, such as *Emys orbicularis* [36], *Dermochelys coriacea* [37], *Malaclemys terrapin* [38], *Chelydra serpentina* [39], *T. scripta* [40] and *Chrysemys picta* [41]. Since aromatase expression increases in the female gonad, we expected even a mild upregulation after *Sox9* knockdown, suggesting a possible feminization of the gonads. However, aromatase expression levels did not significantly change in gonads treated with the three different doses of siRNA (Figure 4C); instead, they remained low, as in the *in ovo* control incubated at MPT (st 23) and experimental controls, CO, CL and CS. This is consistent with the absence of morphological effects, since the role of aromatase has been linked to ovarian differentiation. As explained above, this could be due to the short time the gonads were maintained in culture (72 hours). It is known that in *L. olivacea*, the TSP at MPT lasts approximately six days [23], and also, it has been shown that in several turtles, aromatase upregulation is presented at the end of the TSP at FPT [36,37,38,39,40,41]. Thus, in the present work, it was not possible to reach the onset of aromatase upregulation; therefore, the potential effect of *Sox9* silencing on aromatase expression in cultured gonads was not detected.

It has been previously shown that female specific genes, such as *FoxL2*, are upregulated after *Sox9* knockdown in mouse gonads maintained in culture for three days [42]; moreover, it is known that aromatase is positively regulated by FOXL2 in the fetal goat ovary [43]. It is likely that in reptiles, a similar gene network is participating in the developing ovary, where a repression of *Sox9* could lead to a concomitant upregulation of *FoxL2* and aromatase; therefore, it is recommended to maintain the gonads in culture for a longer period of time (evaluating at the same time the duration of the silencing effect), taking into account the slow rate of development that has been shown in *L. olivacea* [20].

## 4. Conclusions

We have established a gene-specific knockdown method to investigate if SOX9 is required for testis determination/differentiation; this question remains to be fully answered, since gonads need to be maintained in culture for a longer period of time in order to differentiate, and expression of female-specific genes should be measured. Nevertheless, we were able to measure *Amh* expression under a *Sox9* knockdown environment. The regression of the Müllerian duct by AMH is an important event in the male reproductive system differentiation pathway. This first report of *Sox9* gene silencing by RNAi in a gonad culture system of turtles displaying TSD shows a coordinated expression of both genes, supporting the hypothesis that SOX9 directly or indirectly regulates *Amh*. Although possible off-target effects and the viability of the transfected organs remain to be further investigated, the demonstrated efficacy of this technique in turtle gonadal cultures expands our toolbox to address critical questions of sex determination pathways in TSD.
